# HubZIP12-mediated transcriptional activation of betalain biosynthetic genes enhances pigmentation in pitaya (*Hylocereus*)

**DOI:** 10.1186/s43897-026-00243-w

**Published:** 2026-10-08

**Authors:** Irfan Ali Sabir, Xinglong Hu, Jiayi Chen, Yonghua Qin

**Affiliations:** Guangdong Provincial Key Laboratory of Postharvest Science of Fruits and Vegetables/Key Laboratory of Biology and Genetic Improvement of Horticultural Crops (South China), Ministry of Agriculture and Rural Affairs, College of Horticulture, South China Agricultural University, Guangzhou, 510642 China

Pitaya, a beloved tropical fruit, is classified within the Cactaceae family of the Caryophyllales order and comprises species from the *Hylocereus* and *Seleniereus*. The captivating pitaya fruit is distinguished by its peel and pulp colors, leading to the categorization of three main species: *H. megalanthus* or *S. megalanthus* (yellow peel with white pulp), *H. monacanthus* or *H. polyrhizus* (red peel with red pulp), and *H. undatus* (red peel with white pulp) (Shah et al. 2023). Conspicuously, pitaya stands out in commercial fruit cultivation for its high betalain content, unique compounds that attracts consumers (Hu et al. 2025). Betacyanins and betaxanthins are two primary forms of water-soluble, nitrogen-containing compounds that originate from tyrosine. Betacyanins are purple-red in color, while betaxanthins are yellow. The differential biosynthesis and accumulation of betalains contribute to the distinct pigmentation patterns between the peel and flesh of pitaya. Current genome-wide analysis of the pitaya genome has revealed 74 *HubZIP* genes, similar to those found in various other plant species, indicating that the size of the bZIP family has been conserved by similar evolutionary processes. Clustering of bZIP proteins in phylogenetic analysis based on the pitaya, *Beta vulgaris*, and *Arabidopsis thaliana* grouped the bZIP proteins into ten clades (Fig. 1A), which are similar to the model plant groupings that have been reported earlier (Dröge-Laser et al. 2018). The presence of members of the pitaya *bZIP* gene in all the clades suggests that diversification of this gene family might have preceded the divergence of species and that many of these transcription factors (TFs) have some conserved biological functions (Fig. S1). Structural characterization of *HubZIP* genes showed great variation in exon–intron structure, including intronless genes and genes with one or more introns (Fig. S2), a trend also evident in other plant species like *Vitis vinifera* (Liu et al. 2014) and *Fragaria vesca* (Wang et al. 2017). Gene structures across the phylogenetic groups differed; however, members of the same clade tended to have similar exon–intron patterns and conserved motif patterns, implying that they might have been formed by the same ancestral genes and then retained the structural pattern through evolution. This observation was further supported in motif analysis (Fig. S3), where the canonical bZIP domain was also found to be highly conserved in all HubZIP proteins, which is vital in DNA-binding activity and transcriptional regulation. Analysis of gene duplication revealed that the main processes contributing to the proliferation of the *HubZIP* gene family were dispersed and segmental compositions in pitaya (Fig. 1B), which is also typical of other plant genomes. The Ka/Ks ratios obtained against pairs of duplicated genes were less than one (Fig. 1B), which implies that these genes have undergone strong purifying selection and their functions have been preserved over the evolutionary period. Comparative synteny analysis further revealed significant collinearity between pitaya *HubZIP* genes and their orthologs in *B. vulgaris* and *A. thaliana* (Fig. S5), reflecting shared evolutionary origins and suggesting that many *bZIP* genes emerged before the divergence of these species. While these bioinformatic analyses provide important insights into the evolutionary history and structural characteristics of the *HubZIP* gene family, functional validation is essential to understand the biological roles of these TFs, particularly in the regulation of pigment biosynthesis in pitaya fruit. *HubZIP* gene promoters were analyzed, indicating that they contained a large number of *cis*-regulatory elements that are related to plant growth, hormone signaling, and stress responses, such as ABRE, CAAT-box, and other regulatory motifs (Fig. S6). The most prevalent of them was the ABRE motif, a motif linked to abscisic acid (ABA) signaling, indicating that ABA-mediated pathways might have played a role in fruit development and pigmentation (Karppinen et al. 2018). ABA signaling is important in regulating fruit ripening and color acquisition in non-climacteric fruits like strawberry (Chai et al. 2011), where ABA receptors regulate pigment accumulation and influence developmental timing in fruit. This evidence indicates that transcriptional networks that can regulate pigment biosynthesis and fruit maturation are likely to involve ABA-responsive TFs, such as bZIP family members. However, pitaya fruit coloration depends mostly on the presence of betalain pigments, which are produced by a pathway of several important structural genes such as *HuADH1*, *HuCYP76AD1-1* and *HuDODA1* (Xie et al. 2024). These structural genes have been reported to be important building blocks of betalain biosynthesis, although the transcriptional regulators that regulate these genes have not been comprehensively described. Transcriptomic (Figs. S7, S8, S9, S10) and qRT-PCR results indicated that several *HubZIP* genes develop dynamic patterns of expression throughout pitaya fruit development (‘Honghuaqinglong’ and ‘Guanhuabai’) and that a portion of *HubZIP* genes would peak their expression during the coloration phase (Fig. S11). *HubZIP12* showed significant upregulation at the pigmentation stage, indicating that it may be a key transcriptional regulator of betalain biosynthesis. Moreover, a virus-induced gene silencing (VIGS) assay targeting *HubZIP12* in pitaya fruit was conducted to validate this hypothesis experimentally. Silencing of *HubZIP12* resulted in a clear reduction in peel redness and a considerable drop in betacyanin accumulation, whereas betaxanthin levels remained predominantly unchanged (Fig. 1F). These phenotypic changes were also accompanied by a significant reduction in the levels of *HuCYP76AD1-1* and *HuDODA1* transcription, suggesting that HubZIP12 is an upstream regulator of these structural genes whose expression is positively regulated by HubZIP12. HubZIP12 is exclusively localized in the nucleus (Fig. 1D), consistent with its proposed role as a TF in gene regulation. Transcriptional activity assays conducted in yeast and in *Nicotiana benthamiana* transient-expression systems confirmed that HubZIP12 is a transcriptional activator capable of inducing downstream gene expression (Fig. 1C). The dual-luciferase reporter assays also supported this regulatory relationship, in which HubZIP12 significantly increased the promoter activity of *HuCYP76AD1-1* and *HuDODA1* (Fig. 1C). In addition, the direct interaction between HubZIP12 and the promoters of these structural genes was verified by yeast one-hybrid assays (Fig. 1E). To further confirm these interactions on the biochemical level, electrophoretic mobility shift assays (EMSA) were conducted on purified recombinant HubZIP12 protein. These findings confirmed HubZIP12 to be a direct transcriptional regulator of genes in betalain biosynthesis by binding to ACGT motifs, which are present in the promoter of *HuCYP76AD1-1* and *HuDODA1* (Fig. 1E). The potential of HubZIP12 to regulate multiple structural genes indicates that it could be a central regulatory node orchestrating metabolic flux in the betalain biosynthetic pathway. This type of coordinated transcriptional regulation is a hallmark of secondary metabolite pathways in plants, in which TFs integrate developmental signals and environmental inputs to regulate metabolite biosynthesis. These molecular experiments provide strong functional evidence that HubZIP12 is a key regulator of betalain accumulation in pitaya fruit (Fig. 1G). However, the regulatory network governing pigment biosynthesis is likely complex and may incorporate multiple TF families, such as MYB, bHLH, and NAC. Further studies using genetic transformation, chromatin immunoprecipitation sequencing, and protein–protein interaction studies will be needed to understand better the larger regulatory framework that controls betalain biosynthesis in pitaya. These molecular mechanisms will be valuable not only for advancing our understanding of the mechanisms that control pigment responses in betalain-producing plants but also as targets for molecular breeding strategies aimed at improving fruit color and nutritional quality.

**Fig. 1:**
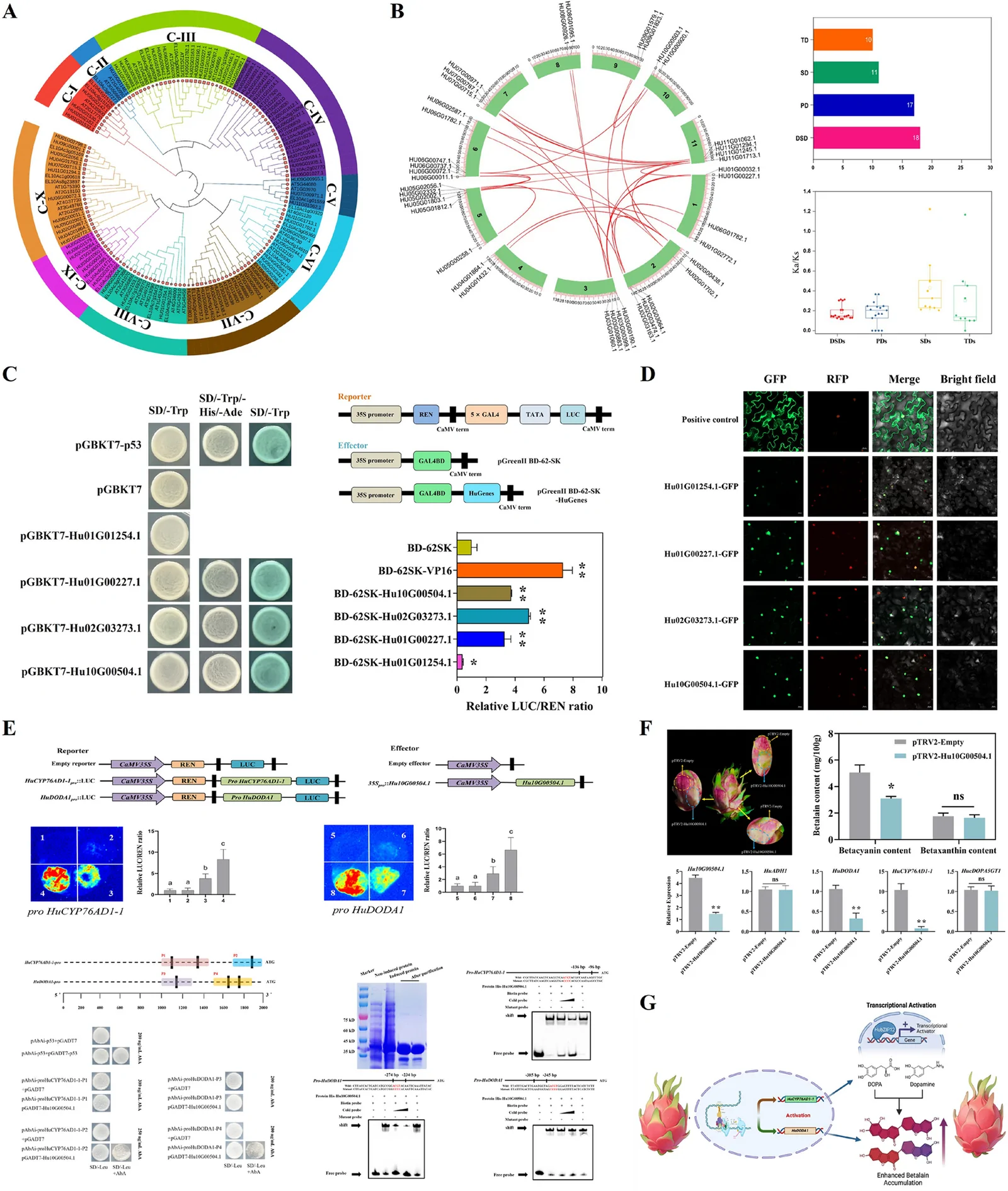
Working model of functional evidence of HubZIP12 for betalain biosynthesis in pitaya. **A** Maximum likelihood (ML) phylogenetic tree of bZIP proteins. **B** Chromosomal distribution (Fig. S4) of duplicated gene pairs associated by red lines. Frequency distribution of duplication events. Selection pressure analysis showing Ka/Ks ratios for each duplication type. **C** Transcriptional activation analyses. **D** Transient expression localization of HubZIP-GFP fusion proteins in *Nicotiana benthamiana* leaf cells. **E** HubZIP12 promotes the expression of *HuCYP76AD1-1* and *HuDODA1* by directly binding to their promoters. **F** Functional characterization of *HubZIP12* (*Hu10G00504.1*) in pitaya fruit. **G** Proposed regulatory mechanism of HubZIP12 in modulating betalain biosynthesis in pitaya. The protein significantly influences the upregulation of structural genes involved in betalain biosynthesis, such as *HuCYP76AD1-1* and *HuDODA1*. The two genes are activated through direct binding of HubZIP12 to their promoter regions, thus facilitating the biosynthesis of betalains in pitaya

## Supplementary Information

Supplementary Material 1.

Supplementary Material 2.

Supplementary Material 3.

## Data Availability

RNA-Seq data from fruit developmental stages of both 'Honghuaqinglong' (29, 32, 35, and 38 DAP) and 'Guanhuabai' (26, 29, 32, and 35 DAP) cultivars have been deposited in the NCBI SRA database under accession number PRJNA1082039.

## References

[CR1] Chai YM, Jia HF, Li CL, Dong QH, Shen YY. FaPYR1 is involved in strawberry fruit ripening. J Exp Bot. 2011;62:5079–89. 10.1093/jxb/err207 21778181

[CR2] Dröge-Laser W, Snoek BL, Snel B, Weiste C. The Arabidopsis bZIP transcription factor family-an update. Curr Opin Plant Biol. 2018;45:36–49. 10.1016/j.pbi.2018.05.001 29860175

[CR3] Hu HM, Pradhan N, Xiao JB, Xia R, Liao P. Chromatic symphony of fleshy fruits: functions, biosynthesis and metabolic engineering of bioactive compounds. Mol Hortic. 2025;5:19. 10.1186/s43897-024-00142-y PMC11963455 40170175

[CR4] Karppinen K, Tegelberg P, Häggman H, Jaakola L. Abscisic acid regulates anthocyanin biosynthesis and gene expression associated with cell wall modification in ripening bilberry (*Vaccinium myrtillus* L.) fruits. Front Plant Sci. 2018;9:406326. 10.3389/fpls.2018.01259 PMC6124387 30210522

[CR5] Liu J, Chen N, Chen F, Cai B, Santo SD, Tornielli GB, et al. Genome-wide analysis and expression profile of the bZIP transcription factor gene family in grapevine (*Vitis vinifera*). BMC Genomics. 2014;15:1–18. 10.1186/1471-2164-15-281 PMC4023599 24725365

[CR6] Shah K, Chen JY, Chen JX, Qin YH. Pitaya nutrition, biology, and biotechnology: a review. Int J Mol Sci. 2023;24:13986. 10.3390/ijms241813986 PMC10530492 37762287

[CR7] Wang XL, Chen X, Yang TB, Cheng Q, Cheng ZM. Genome-wide identification of bZIP family genes involved in drought and heat stresses in strawberry (*Fragaria vesca*). Int J Genomics. 2017. 10.1155/2017/3981031. PMC5405593 28487861

[CR8] Xie FF, Shah K, Chen CB, Sabir IA, Chen JY, Chen JX, et al. Unraveling betalain suppression in pitaya: insights from co-activator HuMYB9 binding at *HuCYP76AD1-1*, *HuADH1*, and *HuDODA1* super-enhancers. Food Qual Saf. 2024;8:fyae016.

