# Supplementary material for: HubZIP12-mediated transcriptional activation of betalain biosynthetic genes enhances pigmentation in pitaya (Hylocereus)
Source: Mol Hortic. 2026 Oct 8;6:70. doi: 10.1186/s43897-026-00243-w (PMC13647800; doi:10.1186/s43897-026-00243-w)
Supplement: Supplementary file 1 [file 43897_2026_243_MOESM1_ESM.docx]

**Fig. S1** Anticipated biological processes, molecular functions, cellular components, and subcellular localization of the *HubZIP* gene family were analyzed to gain comprehensive insights into their functional roles within cellular processes and regulatory networks.

**Fig. S2** The gene structures (introns/exons) of pitaya *bZIP* genes. Introns are depicted by gray lines, while exons and untranslated regions (UTRs) are shown in yellow and green, respectively

**Fig. S3** Conserved motifs analysis in the HubZIP transcription factors. The legend, located at the top provides essential details for interpretation

**Fig. S4** chromosomal localization for *HubZIP* gene family

**Fig. S5** The syntenic relationship between *bZIP* genes in *H. undatus*, *A. thaliana* and *B. vulgaris* is depicted. Syntenic gene pairs are connected by red lines, highlighting their conserved genomic locations

**Fig. S6** *Cis*-regulatory elements in *HubZIP* gene family

**Fig. S7** Transcriptome analysis was conducted to evaluate the expression patterns of *HubZIP* genes across various fruit developmental stages of pitaya. A, Four fruit developmental stages were examined for 'HHQL' (S1: 29 DAP, S2: 32 DAP, S3: 35 DAP, S4: 38 DAP); B, 'GHB' (S1: 26 DAP, S2: 29 DAP, S3: 32 DAP, S4: 35 DAP) pitayas.

**Fig. S8** Tissue-specific expression profiling of *HubZIP* genes was conducted via RNA-Seq analysis, encompassing vegetative tissues and four developmental phases of flower organs in pitaya.

**Fig. S9** Transcriptomic profiling revealed tissue-specific *HubZIP* expression patterns, including dynamic changes across three fruit developmental stages (29, 35, and 49 DAF)

**Fig. S10** Transcriptome analysis in *HubZIP* gene family

**Fig. S11** The expression levels of 18 genes were analyzed using qRT-PCR across four fruit developmental stages. These stages were examined for both ‘HHQL’ (S1: 29 DAP, S2: 32 DAP, S3: 35 DAP, S4: 38 DAP) and ‘GHB’ (S1: 26 DAP, S2: 29 DAP, S3: 32 DAP, S4: 35 DAP) pitayas. The standard deviations for the three replicates are shown by the error bars.
