# Supplementary material for: HubZIP12-mediated transcriptional activation of betalain biosynthetic genes enhances pigmentation in pitaya (Hylocereus)
Source: Mol Hortic. 2026 Oct 8;6:70. doi: 10.1186/s43897-026-00243-w (PMC13647800; doi:10.1186/s43897-026-00243-w)
Supplement: Supplementary file 2 [file 43897_2026_243_MOESM3_ESM.docx]

**Material & methods**

**Plant material**

Two pitaya cultivars, 'Honghuaqinglong' (HHQL, green-skinned with white flesh, *Hylocereus stenopterus*) and 'Guanhuabai' (GHB, red-skinned with white flesh, *H. undatus*), along with *Nicotiana benthamiana* were used as experimental materials. The pitaya plants were maintained under controlled horticultural conditions at South China Agricultural University's orchard, where standard cultivation practices were implemented to ensure consistent growth and development. This included regulated irrigation, optimized soil nutrition, and appropriate light management tailored to each cultivar's physiological requirements. The sampling technique included a thorough evaluation of the various phases of fruit growth. Samples of 'HHQL' pitaya were obtained at certain time intervals, including before the coloring phase (S1, 29 d after pollination, DAP), the coloring period (S2, 32 DAP), at the stage of maturity (S3, 35 DAP), and post-maturity (S4, 38 DAP) used for RNA-Seq. Regarding the 'GHB' pitaya variety, samples were obtained at four different stages of fruit development to conduct a comprehensive gene expression study. These stages include 26 DAP before the coloring phase (S1), 29 DAP during the coloring phase (S2), 32 DAP at maturity (S3), and 35 DAP post-maturity (S4). To prepare the samples for further analysis, they were promptly frozen in liquid nitrogen and stored at -80 ℃. For VIGS assays, scale tissues from 'GHB' pitaya at 23 DAP were utilized. *N. benthamiana* served as the model system for subcellular localization.

**Unveiling the Compilation, Identification, and Molecular Profiling of *bZIP* Genes in pitaya**

The *bZIP* genes were annotated and identified in the pitaya genome using a multi-step process. Initially, the genomic sequences of pitaya were acquired from the website http://www.pitayagenomic.com/ for analysis (Chen et al., 2021, 2022a, 2025). Protein sequence alignments for PF00170 (bZIP TF), PF07716 (basic region leucine zipper), and PF03131 (bZIP MAF TF) were downloaded in the Stockholm format, and an alignment-based model was constructed using Hmmbuild. The Hmmsearch tool was then employed to search for elected genes against the whole pitaya genome database. Furthermore, a set of 74 protein sequences from *A. thaliana* were utilized as queries in Blastp, with an E-value threshold of <1 × 10^-5^, to identify putative bZIP protein sequences in pitaya. The BioEdit software was used for this analysis. The bZIP protein sequences were aligned and deleted any duplicated genes to make sure they were accurate. The discovered *bZIP* genes were subjected to a rigorous validation process to guarantee accuracy and dependability. This involved the utilization of esteemed resources such as SMART (http://smart.emblheidelberg.de) (Letunic et al., 2012), Pfam database (http://pfam.xfam.org) (Finn, 2006), and InterProScan (http://www.ebi.ac.uk/interpro/sea) (Finn, 2006; Letunic et al., 2012; Zdobnov and Apweiler, 2001). Crucial molecular features were predicted to further characterize the *bZIP* genes. The ExPASY server (http://web.expasy.org/protparam) was key in estimating parameters such as molecular weight (MW), number of amino acids, and isoelectric point (IP). Additionally, the WoLF PSORT program (http://www.genscript.com/wolf-psort.html) was used to anticipate the subcellular localization of the *bZIP* genes (Horton et al., 2007).

**Phylogenetic Investigation of the *bZIP* Gene Family: An Analysis of Evolutionary Relationships**

Full-length bZIP protein sequences from pitaya have been assembled utilizing ClustalX, employing pairwise deletion and 1000 bootstraps for robustness. The phylogenetic tree was developed using the Molecular Evolutionary Genetics Analysis (MEGA) software (Thompson et al., 1997) via the neighbor-joining (NJ) method (JTT model, 1000 bootstraps) (Tamura et al., 2011). Additionally, the online version of the IQ-TREE software was employed to generate a maximum likelihood (ML) phylogenetic tree, which was an alternative approach. (http:/iqtree.cibiv.univie.ac.at). The iTOLs program (http://itol.embl.de) was used to show the phylogenetic trees, enabling an interactive and useful visualization of the trees (Letunic and Bork, 2019).

**Gene Structure and Conserved Motif Analysis**

Several tools were employed for visualization and analysis to gain deeper insights into the structural characteristics of the *bZIP* genes. The Gene Structure and Display server 2.0 (GSDS; http://gsds.cbi.pku.edu.cn/Gsds_abou.php) played a pivotal role in visualizing key features such as the position and composition of exons and introns, as well as binding sites and conserved elements (Abdullah et al., 2018; Hu et al., 2015). By utilizing this web-based platform, the intricate gene structure was effectively portrayed, enabling a comprehensive understanding of the organization and arrangement of exonic and intronic regions. Furthermore, the identification and visualization of conserved motifs within the *bZIP* genes were accomplished using the Multiple Em for Motif Elicitation (MEME) software (http://meme-suite.org). By employing this tool, conserved motifs were elucidated; shedding light on potential regulatory elements and functional characteristics associated with the *bZIP* genes (Bailey et al., 2009; Manzoor et al., 2021). This facilitated a deeper exploration and analysis of the underlying genetic components and regulatory mechanisms governing the *bZIP* gene family.

**Investigating Chromosomal Distribution Patterns and Conserved Domains**

A comprehensive approach utilized pitaya genome databases to obtain precise positional information for all *bZIP* genes (Chen et al., 2025). The obtained data were meticulously processed and visualized using the Mapinspect software (http://mapinspect.software.com) (Jung et al., 2004; Sneddon et al., 2012). An integrated approach was employed to identify conserved domains within bZIP proteins. The HMMER software (http://hmmer.org/), protein family's database (Pfam) and CDART (Conserved Domain Architecture Retrieval Tool) were used to detect and analyze the conserved domains with high precision and reliability (Geer et al., 2002; Johnson et al., 2010; Mistry et al., 2021). These computational tools provided valuable insights into the conserved structural motifs present in bZIP proteins, further enhancing our understanding of their functional characteristics and evolutionary relationships.

***Cis*-elements Analysis in Pitaya**

The promoter sequences of pitaya *bZIP* genes were methodically analyzed by examining a region of 1500 bp located upstream of the initiation codon. This region was carefully selected, as it often encompasses critical regulatory elements responsible for gene expression. The PlantCARE web tool (http://bioinformatics.psb.ugent.be/webtools/plantcare) was utilized to predict and screen for various *cis*-elements within the identified promoter sequences (Lescot, 2002). In the pitaya, the discovery of these *cis*-elements led to a more in-depth comprehension of the transcriptional control of the *bZIP* genes as well as the probable functional importance of these genes.

**Analysis of Gene Duplication Patterns, Collinearity Relationships, and Ka/Ks Ratios: Insights into Genetic Evolution**

The Multiple Collinearity Scan (MCScanX) toolkit was utilized in conjunction with BLASTP (E <1 × 10^−5^) against the pitaya genome to conduct collinearity analysis. This comprehensive approach facilitated the identification of various modes of gene duplications, including whole-genome duplication (WGD)/segmental, dispersed, proximal, and tandem duplications in pitaya. The MCScanX toolkits, known for their reliability, were employed for this purpose (Qiao et al., 2015; Wang et al., 2012). To visualize the gene duplications and collinearity relationships, state-of-the-art software such as TBtools and Circos was employed. These techniques facilitated the design of aesthetically pleasing visualizations, offering a thorough overview of gene duplications and associated collinearity patterns (Chen et al., 2020). Key parameters such as the nonsynonymous (Ka) and synonymous (Ks) mutation rates were obtained to investigate the evolutionary dynamics of the duplicated gene pairs. The Plant Genome Duplication Database (PGDD) was a reliable resource for obtaining these rates, providing comprehensive information on gene duplication events (http://chibba.agtec.uga.edu/duplication). The Ka/Ks ratio, derived from these mutation rates, offered valuable insights into the selective pressures and evolutionary forces acting on duplicated genes (Lee et al., 2013). For multiple alignments and Ka/Ks ratio calculations, the MAFFT software and associated calculators were employed. These tools ensured accurate and reliable analysis, providing a deeper understanding of the evolutionary dynamics and functional divergence of duplicated gene pairs (<https://github.com/qiaoxin/Scripts_for_GB/tree/master/calculate_Ka_Ks_pipeline>) (Qiao et al., 2019; Wang et al., 2010).

**Unraveling Gene Expression Profiles: RNA Extraction, Reverse Transcription, and qRT-PCR Analysis**

Transcriptome data were obtained from various fruit developmental stages of 'HHQL' at 29 DAP, 32 DAP, 35 DAP, and 38 DAP. Regarding the 'GHB' variety, stages of 26 DAP, 29 DAP, 32 DAP, and 35 DAP (accession number PRJNA1082039) were obtained from the National Center for Biotechnology Information (NCBI) database at https://www.ncbi.nlm.nih.gov/. These data were utilized to visualize the expression patterns of *HubZIPs*, and the HeatMap program within the TBtools software was employed for this purpose.

Total RNA of all fruit samples were extracted utilizing the RNAprep Pure Plant Kit (Tiangen Biotech Co. Ltd., Beijing). The PrimeScript^TM^ RT reagent Kit with gDNA Eraser (TaKaRa) was used in conjunction with a quantity of 1.0 μg of RNA to perform reverse transcription. The Beacon Designer 7.9 program was used for primer design (Table S2). Quantitative real-time PCR (qRT-PCR) was performed according to a previously described methodology (Chen et al., 2022b)

using the SYBR premix *ExTaq* II. The *Actin(1)* gene was employed as an internal reference control to normalize the expression data (Chen et al., 2019). The corresponding expression level of the *bZIP* genes was determined by the 2^−ΔΔC^T method (Livak and Schmittgen, 2001). All reactions were performed in triplicate to ensure technical reproducibility.

**Subcellular Localization Analyses**

The complete coding sequences (CDS) of *HubZIP12*, *HubZIP13*, *HubZIP14*, and *HubZIP15*, excluding the termination codon, were integrated into the pGreen-35S-GFP vector to create recombinant plasmids (primers are provided in Table S2). Subsequently, these recombinant constructs, namely pGreen-35S-HubZIP12-GFP, pGreen-35S-HubZIP13-GFP, pGreen-35S-HubZIP14-GFP, and pGreen-35S-HubZIP15-GFP were individually introduced into *Agrobacterium tumefaciens* strain GV3101 (pSoup-p19) (Shanghai Weidi Biotechnology Co. Ltd., China). Concurrently, a control vector, pGreen-35S-GFP, was also prepared. For transient expression assays, *A. tumefaciens* cultures containing the target constructs were co-infiltrated into *N. benthamiana* leaves along with At1g22590-RFP as a nuclear localization marker. Following a 48 h incubation period under controlled growth conditions, infiltrated leaf tissues were harvested for microscopic analysis. Subcellular localization patterns were examined using a confocal microscope LSM800 (Zeiss. Oberkochen, Germany), with simultaneous detection of GFP fluorescence (indicating target protein expression) and RFP signals (marking nuclear compartments). This co-localization approach enabled precise determination of protein subcellular distribution (Lee et al., 2012).

**Transcriptional Activity Analyses and Dual-luciferase Transient Expression Assays**

The ORFs of *HubZIP12, HubZIP13, HubZIP14*, and *HubZIP15* were directionally cloned into the pGBKT7 bait vector. For control experiments, we co-transformed positive control plasmids (pGBKT7-p53) and negative control pairs (bait constructs + empty pGBKT7). All constructs were transformed into Y2HGold yeast competent cells and initially selected on SD/-Trp dropout medium at 30 ℃ until colonies formed. For interaction testing, a single colony was resuspended in 0.9% NaCl and plated onto stringent selection media (SD/-Trp/-His/-Ade containing Kan) supplemented with 20 μg/mL X-α-gal. Plates were incubated at 30 ℃ for 3-5 d to assess both growth and α-galactosidase activity, with positive interactions indicated by blue colony formation on the quadruple selection medium. Transcriptional activation capability of HubZIP12, HubZIP13, HubZIP14, and HubZIP15 was evaluated according to color changes of the yeast colonies, indicative of protein-protein interactions.

To evaluate the transcriptional activation potential of HubZIP12, transient expression assays were performed in *N. benthamiana* leaves using a dual-luciferase reporter system. The full-length CDS of *HubZIP12* was cloned into the pGreenII BD-62-SK vector to generate the effector construct. For reporter activity detection, we used pGreenII 0800-LUC containing a synthetic promoter consisting of five tandem GAL4 binding elements (5×GAL4) upstream of the minimal CaMV 35S TATA box driving the firefly luciferase (LUC) gene. This experimental design allowed specific measurement of HubZIP12’s ability to activate transcription through the GAL4 binding sites, with Renilla luciferase serving as an internal control for normalization. pGreenII BD-62-SK-HubZIP12, negative (BD-62-SK) and positive (BD-62-SK-VP16) controls, along with the reporter vector pGreenII 0800-LUC, were transformed into *A. tumefaciens* GV3101 (pSoup-p19). Effectors and reporters were co-infiltrated into *N. benthamiana* leaves at a 9:1 ratio. After 72 h, LUC/REN ratios were measured using a dual-luciferase assay kit (Promega, USA). *Cis*-elements in *HuCYP76AD1-1* and *HuDODA1* promoters were predicted using PlantCARE. To validate HubZIP12’s binding to these promoters, we employed a dual-luciferase reporter system in *N. benthamiana*. The ~1.5 kb promoter regions of *HuCYP76AD1-1* and *HuDODA1* were cloned into pGreenII 0800-LUC to generate reporter constructs, while, the full-length *HubZIP12* CDS was cloned into pGreenII 62-SK as the effector. For transient assays, effector and reporter constructs were co-transformed into *A. tumefaciens* GV3101 (pSoup-p19) at a 9:1 ratio. Agroinfiltrated leaves were harvested after 72 h post-infiltration, and dual-luciferase activities were quantified using the Firefly/Renilla Luciferase Assay Kit (Promega) according to the manufacturer’s protocol. Three biological replicates were analyzed to ensure reproducibility.

**Virus-induced gene silencing (VIGS) Analyses**

The complete sequence of *HubZIP12* was incorporated into the pTRV2 vector. Following this, the constructs of pTRV1, pTRV2, and pTRV2-HubZIP12/13/14/15 were transformed into the *A. tumefaciens* strain GV3101 (primers are listed in Supplementary Table S2). The bacterial cells were then suspended in an MMA buffer (10 mM MES, 10 mM MgCl_2_, and 100 µM acetosyringone) until the optical density (OD_600_) is 0.4, maintaining a 1:1 ratio in the mixture. The methodologies for VIGS was performed according to our previous studies (Xie et al., 2024).

**Yeast One-hybrid Assay**

Yeast one-hybrid (Y1H) assays were performed to validate HubZIP12’s binding to the G-box elements in the *HuCYP76AD1-1* and *HuDODA1* promoters. The ~400 bp promoter fragments containing the conserved G-box (CACGTG) motifs were cloned into the pABAi bait vector, while the complete *HubZIP12* CDS was inserted into the pGADT7 activation domain (AD) vector. Following co-transformation of these constructs into the Y1HGold yeast strain, transformed cells were plated on SD/-Leu dropout medium and incubated at 30 ℃ for 3-5 d. Protein-DNA interactions were confirmed by yeast growth on selective media containing 200 ng/mL aureobasidin A (AbA), with empty pABAi + pGADT7-HubZIP12 serving as negative controls. Three independent biological replicates were performed to make consistent interaction patterns.

**Electrophoretic Mobility Shift Assay (EMSA)**

The full-length CDS of *HubZIP12* was cloned into the pET-32a expression vector to produce His-tag recombinant protein. Following induction and purification, the HubZIP12-His fusion protein was used for DNA-binding assays. Biotin-labeled 40 bp probes containing the ACGT motifs from *HuCYP76AD1-1* and *HuDODA1* promoters (Table S2) were commercially synthesized by Sangon Biotech. Co., Ltd (Shanghai). Protein-DNA interactions were analyzed following the previously established protocol (Hellman and Fried, 2007).

**Statistical Analysis**

The data were examined using Analysis of Variance (ANOVA), followed by pairwise comparisons using a student’s t-test with a significance threshold of 5%. The statistical analyses were performed using GraphPad version 9.1.1 (GraphPad Software, San Diego, California, USA; www.graphpad.com). The results are presented as the average values along with the standard error of the mean (SEM). Significance levels are denoted as follows: The symbols “*”, “**”, “***”, and “****” were used for a *p*-value less than 0.05, 0.01, 0.001 and 0.0001, respectively. When the *p*-value surpasses 0.05, it is measured as “non-significant” and is represented by the abbreviation “ns”.

Reference

Abdullah, M., Cao, Y., Cheng, X., Meng, D., Chen, Y., Shakoor, A., Gao, J., Cai, Y. The sucrose synthase gene family in Chinese pear (*Pyrus bretschneideri* Rehd.): Structure, expression, and evolution. Molecules 2018; 23, 1-16.

Bailey, T.L., Boden, M., Buske, F.A., Frith, M., Grant, C.E., Clementi, L., Ren, J., Li, W.W., Noble, W.S. MEME SUITE: tools for motif discovery and searching. Nucleic Acids Res. 2009; 37:W202-208.

Chen, C.J, Chen, H., Zhang, Y., Thomas, H.R., Frank, M.H., He, Y.H, Xia, R. TBtools: an integrative toolkit developed for interactive analyses of big biological data. Mol. Plant 2020; 13: 1194-1202

Chen, C.B., Li, F.P., Xie, F.F., Chen, J.X., Hua, Q.Z., Chen, J.Y., Wu, Z.J., Zhang, Z.K., Zhang, R., Zhao, J.T., Hu, G.B., Chen, J.Y., Qin, Y.H. Pitaya Genome and Multiomics Database (PGMD): a comprehensive and integrative resource of *Selenicereus undatus*. Genes, 2022a; 13, 745.

Chen, C.B., Xie, F.F., Shah, K., Hua, Q.Z., Chen, J.Y., Zhang, Z.K., Zhao, J.T., Hu, G.B., Qin, Y.H. Genome-Wide Identification of WRKY Gene Family in Pitaya Reveals the Involvement of HmoWRKY42 in Betalain Biosynthesis. Int.J. Mol. Sci. 2022b; 23, 10568.

Chen, C.B., Wu, J.Y., Hua, Q.Z., Noemi, T.Z., Xie, F.F., Zhang, Z.K., Chen, J.Y., Zhang, R., Hu, G.B., Zhao, J.T., Qin, Y.H. Identification of reliable reference genes for quantitative real-time PCR normalization in pitaya. Plant Methods, 2019; 15: 70.

Chen, J.X., Li, F.P., Liu, J., Mao, Y.C., Gan, Z., Hu, H., Sabir, I.A., Khan, I., Chen, J.Y., Chen, C.B., Zhang, Z.K., Zhao, J.T., Hu, G.B., Wang, S.K., Qin, Y.H. Gap-free genome and efficient transcript purification system reveals the genomes diversity and chlorophyll degradation mechanism in pitaya. J. Integr. Plant Biol. 2025; 67: 1771-1786.

Chen, J.Y., Xie, F.F., Cui, Y.Z., Chen, C.B., Lu, W.J., Hu, X.D., Hua, Q.Z., Zhao, J., Wu, Z.J., Gao, D., Zhang, Z.K., Jiang, W.K., Sun, Q.M., Hu, G.B., Qin, Y.H. A chromosome-scale genome sequence of pitaya (*Hylocereus undatus*) provides novel insights into the genome evolution and regulation of betalain biosynthesis. Hortic. Res. 2021; 8: 154.

Finn, R.D. Pfam: clans, web tools and services. Nucleic Acids Res. 2006; 34, D247-D251.

Geer, L.Y., Domrachev, M., Lipman, D.J., Bryant, S.H. CDART: Protein homology by domain architecture. Genome Res. 2002; 12, 1619-1623.

Hellman, L.M., Fried, M.G. Electrophoretic mobility shift assay (EMSA) for detecting protein–nucleic acid interactions. Nature Protocols, 2007; 2, 1849-1861.

Horton, P., Park, K.J., Obayashi, T., Fujita, N., Harada, H., Adams-Collier, C., Nakai, K. WoLF PSORT: protein localization predictor. Nucleic Acids Res. 2007; 35: W585-W587.

Hu, B., Jin, J.P, Guo, A.Y., Zhang, H., Luo, J.C., Gao, G. Genome analysis GSDS 2.0: an upgraded gene feature visualization server. Bioinformatics, 2015; 31(8): 1296-1297.

Johnson, L.S., Eddy, S.R., Portugaly, E. Hidden Markov model speed heuristic and iterative HMM search procedure. BMC Bioinformatics 2010; 11: 1-8.

Jung, S., Jesudurai, C., Staton, M., Du, Z., Ficklin, S., Cho, I., Abbott, A., Tomkins, J., Main, D. GDR (Genome Database for Rosaceae): Integrated web resources for Rosaceae genomics and genetics research. BMC Bioinformatics 2004; 5: 1-8.

Lee, L.Y.C., Hou, X., Fang, L., Fan, S., Kumar, P.P., Yu, H. STUNTED mediates the control of cell proliferation by GA in Arabidopsis. Development 2012; 139: 1568-1576.

Lee, T.H., Tang, H., Wang, X., Paterson, A.H. PGDD: A database of gene and genome duplication in plants. Nucleic Acids Res. 2013; 41: 1152-1158.

Lescot, M. PlantCARE, a database of plant cis-acting regulatory elements and a portal to tools for in silico analysis of promoter sequences. Nucleic Acids Res. 2002; 30: 325-327.

Letunic, I., Bork, P. Interactive Tree Of Life (iTOL) v4: recent updates and new developments. Nucleic Acids Res. 2019; W1: W256-W259.

Letunic, I., Doerks, T., Bork, P. SMART 7: recent updates to the protein domain annotation resource. Nucleic acids Res. 2012; 40: D302-D305.

Livak, K.J., Schmittgen, T.D. Analysis of relative gene expression data using real-time quantitative PCR and the 2^−ΔΔC^T method. Methods, 2001; 25, 402-408.

Manzoor, M.A., Manzoor, M.M., Li, G.H., Abdullah, M., Han, W., Han, W.L., Shakoor, A., Riaz, M.W., Rehman, S., Cai, Y.P. Genome-wide identification and characterization of bZIP transcription factors and their expression profile under abiotic stresses in Chinese pear (*Pyrus bretschneideri*). BMC Plant Biol. 2021; 21(1):413.

Mistry, J., Chuguransky, S., Williams, L., Qureshi, M., Salazar, G.A., Sonnhammer, E.L.L., Tosatto, S.C.E., Paladin, L., Raj, S., Richardson, L.J., Finn, R.D., Bateman, A. Pfam: The protein families database in 2021. Nucleic Acids Res. 2021; 49(D1): D412-D419.

Qiao, X., Li, M., Li, L.T., Yin, H., Wu, J.Y., Zhang, S.L. Genome-wide identification and comparative analysis of the heat shock transcription factor family in Chinese white pear (*Pyrus bretschneideri*) and five other Rosaceae species. BMC Plant Biol. 2015; 15, 1-16.

Qiao, X., Li, Q.H., Yin, H., Qi, K.J., Li, L.T, Wang, R.Z., Zhang, S.L., Paterson, A.H. Gene duplication and evolution in recurring polyploidization-diploidization cycles in plants. Genome Biol. 2019; 20(38): 1-23.

Sneddon, T.P., Li, P., Edmunds, S.C. GigaDB: Announcing the GigaScience database. GigaScience 2012; 1, 1-2.

Tamura, K., Peterson, D., Peterson, N., Stecher, G., Nei, M., Kumar, S. MEGA5: molecular evolutionary genetics analysis using maximum likelihood, evolutionary distance, and maximum parsimony methods. Mol. Biol. Evol. 2011; 28, 2731-2739.

Thompson, J.D., Gibson, T.J., Plewniak, F., Jeanmougin, F., Higgins, D.G. The CLUSTAL X windows interface: Flexible strategies for multiple sequence alignment aided by quality analysis tools. Nucleic Acids Res. 1997; 25, 4876-4882.

Wang, D.P., Zhang, Y.B., Zhang, Z., Zhu, J., Yu, J. KaKs_Calculator 2.0: a toolkit incorporating gamma-series methods and sliding window strategies. ‌Genom. Proteom. Bioinform‌. 2010; 8, 77-80.

Wang, Y., Wang, X., Paterson, A.H. Genome and gene duplications and gene expression divergence: a view from plants. ‌Ann. N.Y. Acad. Sci. 2012; 1256, 1-14.

Xie, F.F., Shah, K., Chen, C.B., Sabir, I.A., Chen, J.Y., Chen, J.X., Chen, J.Y., Qin, Y.H. Unraveling betalain suppression in pitaya: insights from co-activator HuMYB9 binding at *HuCYP76AD1-1*, *HuADH1*, and *HuDODA1* super-enhancers. Food Qual. Saf. 2024; 8, fyae016.

Zdobnov, E.M., Apweiler, R. InterProScan–an integration platform for the signature-recognition methods in InterPro. Bioinformatics 2001; 17, 847-848.
